# Role of collateral embolization in addition to uterine artery embolization followed by hysteroscopic curettage for the management of cesarean scar pregnancy

**DOI:** 10.1186/s12884-019-2590-2

**Published:** 2019-12-16

**Authors:** Guodong Zhang, Jijun Li, Jun Tang, Lei Zhang, Dechao Wang, Zengtao Sun

**Affiliations:** Department of Interventional Radiology, Shandong Medical Imaging Research Institute Affiliated to Shandong University, Jinan, 250021 People’s Republic of China

**Keywords:** Cesarean scar pregnancy, Collateral, Curettage, Intervention, Uterine artery embolization

## Abstract

**Background:**

The aim of this study was to assess the feasibility, safety and outcome of the embolization of non-gonadal collateral supplying gestational sac (GS) in addition to uterine artery embolization (UAE), followed by hysteroscopic curettage for the management of cesarean scar pregnancy (CSP).

**Methods:**

A retrospective study was undertaken from January 2012 to September 2018 in 24 CSP patients in whom non-gonadal collaterals supplying GS were identified by arterial angiography performed immediately after UAE. These patients underwent attempt collateral embolization in addition to UAE, followed by hysteroscopic curettage for the management of CSP. The 24 patients were divided into two groups based on whether they underwent technically successful collateral embolization (UAE-SCE group) or failed collateral embolization (UAE-FCE group) in addition to UAE. The baseline characteristics and clinical outcomes including time for serum β-human chorionic gonadotropin (β-hCG) levels normalization, blood loss, secondary anemia, and pelvic pain were compared between the two groups. The paired t test and Man Whitney test were used for comparisons of discrete and numerical variables, respectively.

**Results:**

Collateral embolization was techinically successful in 16 (66.7%, 16/24) patients and failed in the other 8 (33.3%, 8/24) patients. There were no significant differences between the two groups in baseline characteristics. The mean blood loss and secondary anemia in the UAE-SCE group were significantly less than UAE-FCE group. No significant difference was found between the two groups in the mean time for β-hCG levels normalization and pelvic pain.

**Conclusions:**

During the management of UAE combined with hysteroscopic curettage for CSP, additional embolization of non-gonadal collateral supplying GS during UAE is feasible and safe in patients with non-gonadal collateral supplying GS, and the additional embolization of the collateral may reduce blood bloss related to hysteroscopic curettage.

## Background

Cesarean scar pregnancy (CSP) is a rare type of ectopic pregnancy defined as a gestational sac (GS) that implants at the site of a previous hysterotomy scar [1]. Currently, the diagnostic criteria of CSP are debated and still not standardized as patient presentations vary considerably, and the diagnosis of CSP made by ultrasound has been widely used.

Considering the risk of complications including uterine rupture, abnormal placentation, invasion into surrounding organs, massive vaginal bleeding and hypovolemic shock, pregnancy termination is generally recommended for patients with diagnosed CSP. In the documented treatment options for the management of CSP, uterine artery embolization (UAE) combined with another techniques including hysteroscopic resection, dilation and curettage, or curettage has been described as an efficient and safe treatment modality. In these combined techniques, the main goal of UAE was to occlude blood flow of bilateral uterine arteries by which to reduce high risk of vaginal hemorrhage related to the following curettage or hysteroscopic resection and accelerate the resolution of the gestational sac [2–4].

Although the bilateral uterine arteries provide the dominant uterine blood supply to uterine fibroid, recruitment of collateral supplying fibroid from the ovarian artery has been well documented in the literature. And it was also documented that failure to recognize and/or embolize the collaterals supplying fibroid during UAE was responsible for substandard clinical result [5, 6].

Similarly, from our experiences in UAE, the GS was supplied by collateral besides gonadal arteries (i.e., bilateral uterine arteries and ovarian arteries) in some CSP cases. To our knowledge, it was not documented the role of the non-gonadal collateral supplying GS in UAE.

Therefore, the purpose of the study was to assess the feasibility, safety and outcome of the embolization of non-gonadal collateral supplying GS in addition to UAE, followed by hysteroscopic curettage for the management of CSP.

## Methods

### Patient selection

Our hospital is the tertiary referral centre providing a range of subspecialty services for more than 60 hospitals in China. From January 2012 to September 2018, 481 patients with diagnosed CSP underwent UAE followed by hysteroscopic curettage in our hospital. In 24 of 481 (5.0%, 24/481) patients, non-gonadal collaterals supplying GS were identified by arterial angiography performed immediately after UAE. These 24 patients underwent attempt collateral embolization in addition to UAE, and were included for retrospective analysis.

This study complies with current ethical consideration and was approved by our institutional ethical committee. The combined procedure of UAE and additional embolization of non-gonadal collateral supplying GS followed by hysteroscopic curettage was explained in full to each patient, and each patient gave an informed consent.

**Fig. 1:**
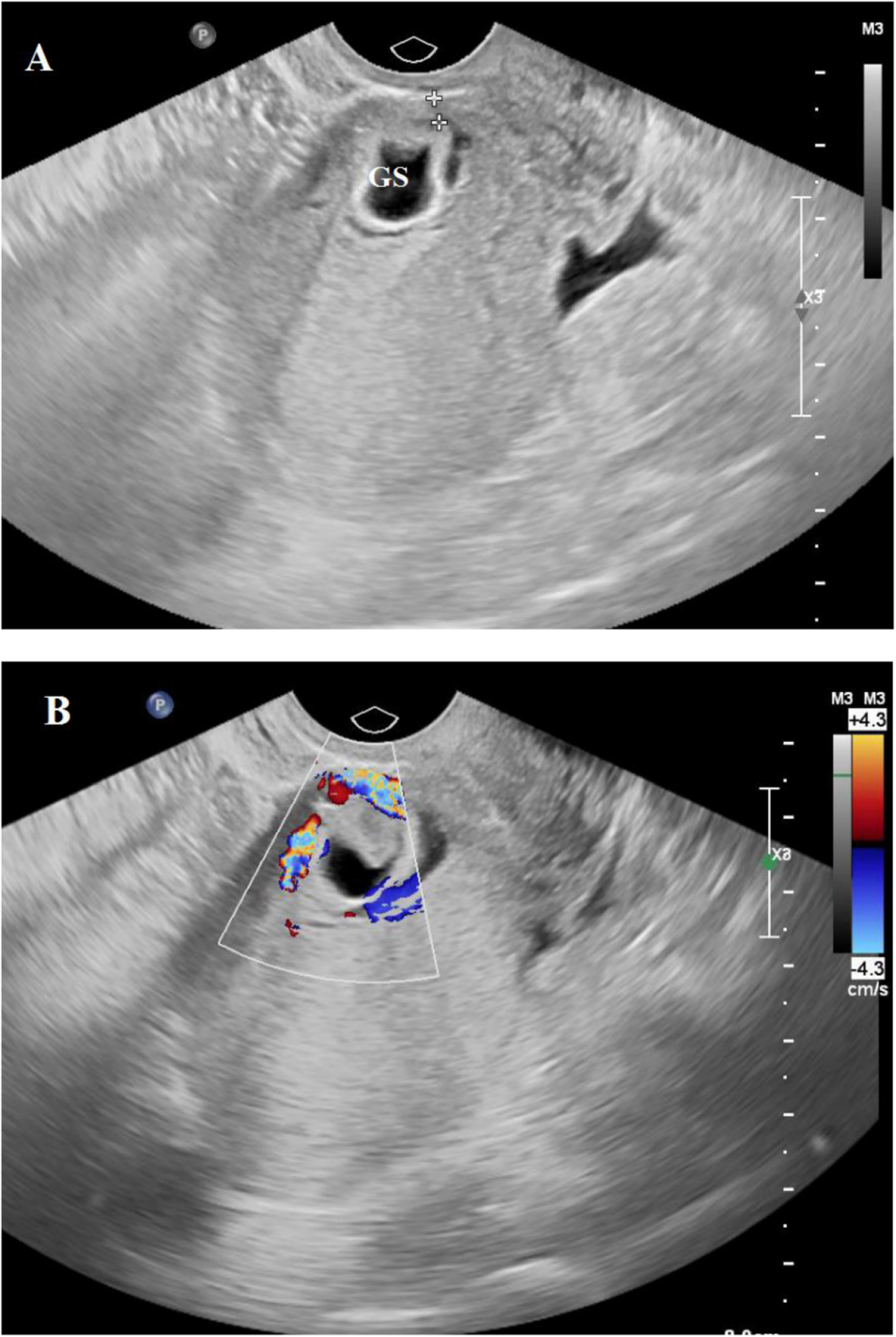
Transabdominal ultrasound (**a**) of a patient with caesarean scar pregnancy showed an empty uterine cavity and cervical canal, and the gestational sac (GS) implanted at the site of a cesarean scar and surrounded by thin myometrium. The Doppler ultrasound (**b**) revealed the increased vascular flow surrounding the GS

The diagnostic criteria for CSP included an increased serum β-human chorionic gonadotropin (β-hCG) level (normal range less than 5 mIU/mL), history of a prior cesarean scar, and ultrasound image that met the criteria proposed by Godin et al. [7], as follows: (i) absence of GS in uterine cavity and cervical canal; (ii) presence of a GS located at the anterior isthmus of uterus with or without cardiac activity; (iii) thinning or absence of myometrium between bladder and the GS (Fig. 1).

All the patients were hospitalized in the department of gynecology. The baseline characteristics of the patients including maternal age, time interval since last cesarean section, gestational age, GS diameter, myometrium thickness (the thickness of the myometrium between the GS and the bladder), and serum β-hCG level were recorded.

### Arterial embolization and hysteroscopic curettage

The patients underwent UAE in our interventional department by two experienced interventional radiologists. Using Seldinger technique, the right common femoral artery access was achieved under local anesthesia. A 5-Fr Cobra catheter (Cordis, USA) was advanced distally to selectively catheterize the left internal iliac artery (IIA) negotiating with a hydrophilic guide-wire. The subsequent digital subtraction angiography (DSA) was performed at the IIA using 16 mL of nonionic contrast medium (Ultravist; Bayer, Germany) at a flow rate of 4 mL/s.

The DSA performed at the IIA was attempt to visualize the vascular anatomy of uterine artery, uterine perfusion, and the hypervascularity enhancement in GS region. When necessary, DSA of the IIA was performed with ipsilateral anterior oblique projection (30°) to reduce the imaging superimpositions and detect the origin of uterine artery. Then, a coaxial microcatheter (Stride 2.6 F, Asahi Intecc or Progreat 2.7 F, Terumo) was introduced coaxially into the uterine artery and advanced distally beyond the origin of the cervicovaginal branch with the digital roadmapping of the IIA. Selective uterine artery angiography was performed to ensure the trajectory of the uterine artery, uterine perfusion, and the hypervascularity enhancement in GS region. The subsequent embolization was performed by injecting the embolic agent slowly under continuous fluoroscopic guidance without reflux to undesired arteries. The embolic agents were 1000–1400 μm sized gelatin sponge particles (Alicon Co. Ltd., Hangzhou, China), which were absorbable agents. Each vial of gelatin sponge particles (1 mL) was mixed with 50 mL solution of contrast medium and saline solution at a 1:1 ratio. Embolization of the uterine artery was performed to the point of complete stasis of blood flow achieved in the ascending uterine artery.

**Fig. 2:**
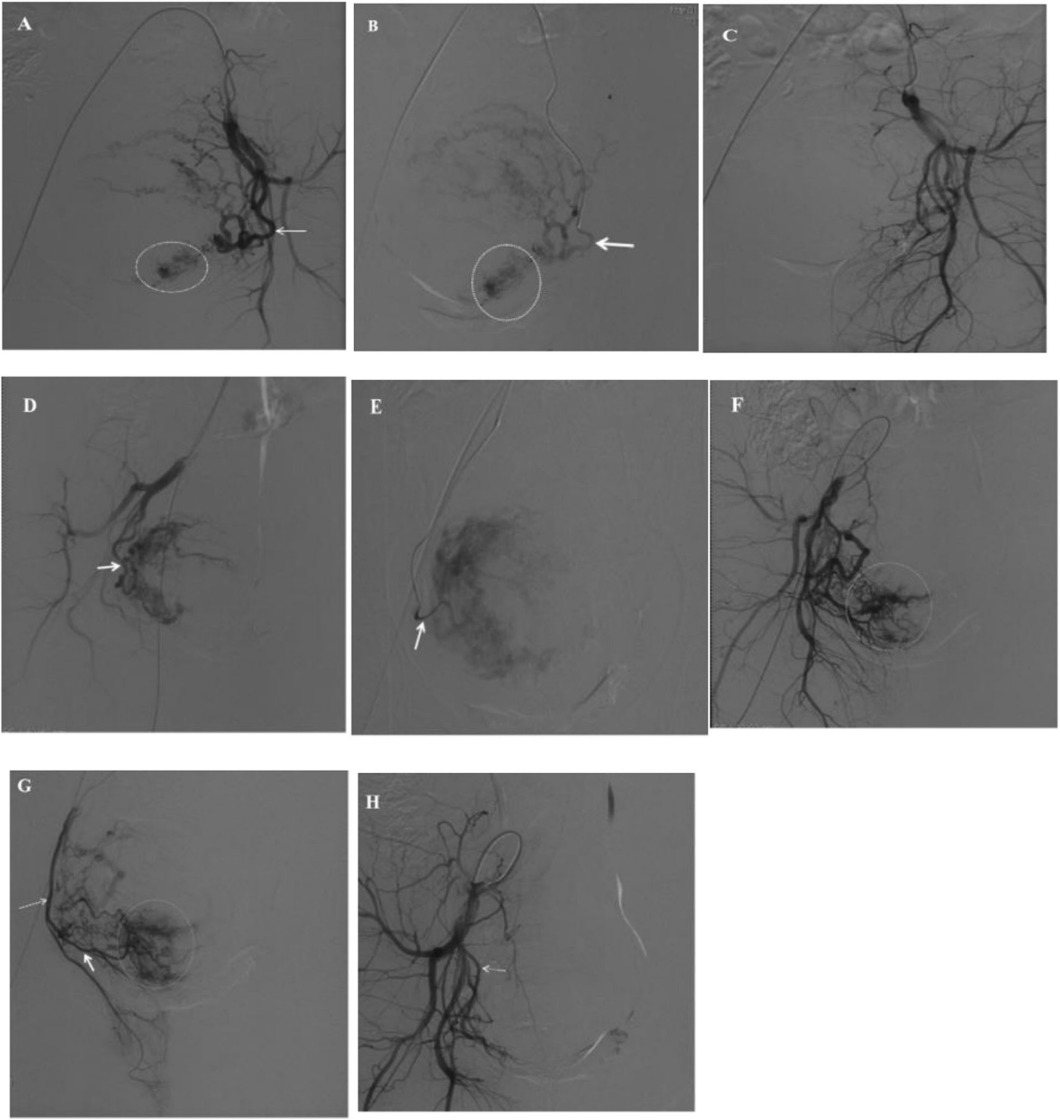
Representative angiographic images of a 32-year-old female patient suffering from cesarean scar pregnancy undergoing embolization of >collateral supplying gestational sac (GS) in addition to uterine artery embolization. Angiography (**a**) of the left internal iliac artery (IIA) demonstrates the dilated left uterine artery (straight arrow) with typical tortuous trajectory and uterine perfusion, and a hypervascular gestational sac (dotted circle). Selective angiography (**b**) following microcatheterization of the left uterine artery (straight arrow) confirms the uterine perfusion and the hypervascular gestational sac (dotted circle). Angiography (**c**) of the left IIA performed after embolization of left uterine artery demonstrates the occlusion of left uterine artery and complete disappearance of uterine perfusion and hypervascularity enhancement in the GS region. Angiography (**d**) of the right IIA performed with ipsilateral anterior oblique projection (30°) demonstrates the dilated right uterine artery with typical tortuous trajectory and marked uterine perfusion. Selective angiography (**e**) following microcatheterization of the right uterine artery confirms the uterine perfusion. Angiography (**f**) of the right IIA performed after embolization of right uterine artery demonstrates the occlusion of right uterine artery, and the marked neovascularity and hypervascularity enhancement in the GS region (dotted circle). Selective angiography (**g**) following microcatheterization indicates the GS is supplied by a collateral (straight arrow) originating from the right internal pudendal artery (dotted arrow). The target collateral shows marked neovascularity and hypervascularity enhancement in the GS region (dotted circle). Angiography (**h**) of the right IIA performed after embolization of the target collateral demonstrates complete disappearance of hypervascularity enhancement in the GS region, without occlusion of the right internal pudendal artery (dotted arrow)

Then postembolization angiongraphy at IIA after the removal of microcatheter was performed immediately to confirm the arterial occlusion of uterine artery and identify the possible presense of collateral supplying GS. The artery with the angiographic appearance of neovascularity and corresponding enhancement in the GS region was interpreted as a collateral supplying GS. Once a collateral supplying GS was identified, further selective microcatheterization and angiography was performed in the collateral to ensure the corresponding enhancement, followed by embolization with aforementioned gelatin sponge particles to the point of complete stasis of blood flow. After the embolization of the left uterine artery and collateral was finished, a Waltman loop was created on the Cobra catheter and the embolization of the right uterine artery and collateral was performed in the same way (Fig. 2). Analgesics and antiemetics were administered when needed.

**Fig. 3:**
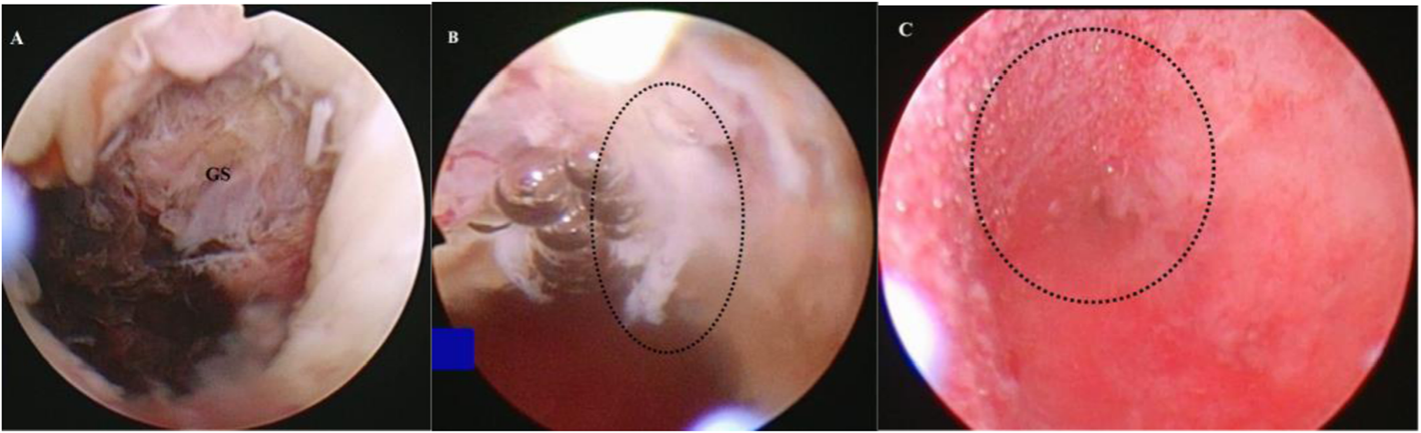
Hysteroscopic management of cesarean scar pregnancy after uterine artery embolization. **a** Before hysteroscopic treatment, the gestational sac (GS) was visualized clearly. **b** After the GS was removed, only residual gestational tissue (dotted circle) was seen. **c** The myometrium (dotted circle) was visualized after the residual gestational tissue was cleared completely using suction curettage

After the embolization, the patient received 100 mg of mifepristone per day for cervical preparation. The following removal of the GS using hysteroscopic curettage technique was conducted by experienced gynecologists within 48 h post-embolization. Under epidural anaesthesia, an operative hysteroscope with a 10-mm external diameter was placed inside the uterus following the cervical dilatation by Hegar dilators. Uterine distension was obtained using 5% glucose solution propelled by a uterine expansion instrument. Subsequently, an operative 26F hysteroscopic resectoscope with an electric wire loop electrode was introduced under transabdominal sonography guidance. The GS was pushed with the wire loop to expose the blood vessel bed of the implantation site. Then, the GS was removed using placenta forceps under direct vision. The following suction curettage with the wire loop was perfomed to clear the residual gestational tissue from the uterine wall. Coagulating the blood vessels with hysteroscopic rolling ball was used to control bleeding if required. This process continued until the gestational tissue was cleared completely and the myometrium was visualized (Fig. 3).

According to the protocols, ultrasonography was performed 3 days after the procedure, and serum β-hCG level was assessed before the procedure and every 3 days after procedure until discharge. Blood count was monitored before the procedure and 24 h after procedure. Patients were discharged when (i) vaginal bleeding ceased or was mild; (ii) steadily decreasing in serum β-hCG levels was observed; and (iii) pelvic pain was absent. Follow-ups after discharge included repeat ultrasonic examination and serum β-hCG level measurement that were performed every 2 weeks until the serum β-hCG level returned to normal, and no residual GS was detected.

### Outcome evaluation

The items evaluations were as follows: anatomic characteristics of the collateral supplying GS, technical success of collateral embolization, and clinical outcomes including time for β-hCG levels normalization and side effects.

Anatomic characteristics of the collateral supplying GS consisting of the origin of each collateral and whether unilateral or bilateral collaterals identified in each patient were recorded. Technical success of collateral embolization was considered when selective embolization in all identified collaterals supplying GS, and complete disappearance of uterine perfusion and hypervascularity enhancement in the GS region on post-embolization angiography at IIA were achieved. Side effects included post-embolization pain, the amount of intraoperative blood loss during curettage, secondary anemia, and whether need for additional blood transfusion or further hysterectomy or laparotomy.

In order to assess the role of the additional collateral embolization, the included patients were divided into two groups based on whether they underwent technically successful collateral embolization (UAE-SCE group) or failed collateral embolization (UAE-FCE group) in addition to UAE. The baseline characteristics and clinical outcomes were compared between the two groups.

### Statistics

The paired t test and Man Whitney test were used for comparisons of discrete and numerical variables, respectively. All statistics were determined using SPSS (SPSS, Chicago, USA) version 22.0 and *P* value of less than 0.05 was considered to be statistically significant.

## Results

### Technical and anatomic result

In the 24 patients included for evaluation, collaterals supplying GS were unilateral in 21 patients and bilateral in 3 patients, resulting in a total of 27 collaterals. As a result, most of the collaterals (81.5%, 22/27) originated from the intern pudental arteries, and the remaining collaterals (18.5%, 5/27) originated from the anterior division of IIAs.

Collateral embolization was techinically successful in 16 (66.7%, 16/24) patients (UAE-SCE group), and failed in the other 8 (33.3%, 8/24) patients (UAE-FCE group) due to the impossibility of selective catheterization because of the angled origins (in 6 patients) or small-sized orfice of the collaterals (in 2 patients).

### Clinical results

The baseline characteristics of the 24 patients were shown in Table 1. There were no significant differences between the two groups in maternal age, gestational age, time interval between last cesarean section and the current pregnancy, GS diameter, the mean serum β-hCG levels, or myometrium thickness.

**Table 1:** Baseline characteristics of the 24 CSP patients with collateral supplying gestational sac

Characteristic	UAE-SCE group (n = 16)	UAE-FCE group (*n* = 8)	*p* value
Maternal age (years)	32.44 ± 5.60	31.13 ± 6.29	0.725
Time interval since last cesarean section (years)	5.50 ± 3.16	4.75 ± 3.20	0.930
Gestational age (days)	54.50 ± 15.00	71.38 ± 7.63	0.136
Gestational sac diameter (mm)	3.61 ± 1.67	5.66 ± 1.02	0.208
Myometrium thickness (mm)	3.11 ± 0.72	3.09 ± 0.86	0.434
Pre-procedural serum β-hCG level (mIU/mL)	58,852.07 ± 12,889.48	59,607.57 ± 23,137.26	0.505

The data are expressed as mean ± standard deviation

*CSP* Cesarean Scar Pregnancy, *UAE* Uterine Artery Embolization, *SCE* Successful Collateral Embolization, *FCE* Failed Collateral Embolization, *β-hCG* β-human Chorionic Gonadotropin

The clinical outcomes after CSP treatments are presented in Table 2. There was no significant difference between the two groups in the mean serum β-hCG levels on post-procedual 3rd day, and the mean time for β-hCG levels normalization. There were no significant differences between the two groups in slight pelvic pain. All the patients with slight pelvic pains recovered quickly after symptomatic treatments.

**Table 2:** Clinical patient outcomes

Characteristic	UAE-SCE group (*n* = 16)	UAE-FCE group (n = 8)	*p* value
Post-procedural serum β-hCG level (mIU/mL)	9296.08 ± 2425.87	8921.17 ± 4182.38	0.740
Time for β-hCG normalization (days)	28.94 ± 1.41	27.88 ± 1.86	0.574
Side effects			
Pelvic pain (n)	3	1	0.705
Amount of blood loss (mL)	78.75 ± 26.63	712.50 ± 110.91	0.025
Anemia (n)	0	3	0.01

The data of pelvic pain and anemia are listed as number, and the other data are expressed as mean ± standard deviation

*UAE* Uterine Artery Embolization, *SCE* Successful Collateral Embolization, *FCE* Failed Collateral Embolization, *β-hCG* β-human Chorionic Gonadotropin

However, the mean blood loss in the UAE-SCE group was significantly less than UAE-FCE group (*P* = 0.025). The number of secondary anemia in the UAE-SCE group was also significantly less than UAE-FCE group (*P* = 0.01). As a result, among the 3 patients with secondary anemia to curettage in the UAE-FCE group, 1 patient received additional treatment with blood transfusion. No hysterectomies or laparotomies were needed for either of the two groups.

## Discussion

The frequency of CSP has increased with the frequency of cesarean section in the recent decade, which might be due to the continuous increase in the cesarean deliveries and the improvement in ultrasound technique which enables early diagnosis of CSP [8]. The reported alternative treatments for CSP included laparotomy, surgical resection, hysteroscopic resection, UAE combined with other techniques including uterine dilation and curettage, systemic or local administration of methotrexate, or hysteroscopic curettage [3]. However, the optimal management for CSP remains unclear.

Although laparoscopic management for CSP has been described with the advantages of a high success rate and acceleration of time for β-hCG levels normalization. Due to the associated risk of massive intraoperative vaginal bleeding which required additional treatments, the use of single hysteroscopic management in terminating CSP is not adequate [9, 10]. Thus, in the present study, UAE was incorporated as a preventive preceding method and performed prior to hysteroscopic curettage for the treatment of CSP. However, massive vaginal bleeding was still be occasionally encountered in patients undergoing the combined techniques. After a retrospective analysis, we found that unsuccessful occlusion of significant collaterals supplying the GS resulted in unsatisfactory clinical results compared with successful occlusion of collaterals. Our finding was similar to previous study that reported nontreatment of the collateral supplying the fibroid during UAE for fibroid maybe associated with the clinical failure [5, 6, 11–13].

In the present study, the collaterals supplying GS originated from the intern pudental arteries, or anterior division of IIAs. To avoid the possible reflux of embolic material and nontarget embolization of intern pudental arteries or other branches of IIAs, the microcatheter was advanced distally in the target collateral before the delivery of embolic material.

The arterial anotomy of pelvis is complex and variant. From our experiences, the use of the ipsilateral anterior oblique projection (30°) in the initial angiography of the IIA is helpful to identify/recognize the origins of the uterine artery, intern pudental artery, and the collateral supplying the GS.

It is challenging to selectively catheterize the collaterals supplying GS. The technical failure of collateral embolization was usually ascribed to the tortuosity or small diameter orfice of the target collaterals, which hampered the selective microcatheterization.

Additionally, UAE has been described to slightly reduce fertility and pregnancy rate which was possibly secondary to ovarian failure resulting from nontarget ovarian embolization via ovarian artery-to-uterine artery anastomoses [14–16]. In the present study, when selective embolization of uterine arteries and collaterals was performed, absorbable and large diameter embolic agents were used, so as to preserve the uterine and avoid nontarget embolization of ovarian arteries as much as possible.

The present study has several limitations. First, it was a retrospective study, so there may have been the possibility of selection bias, and the pre-procedural clinical and imaging data was incomplete. Second, there was only small number of patients included because of the low incidence of collaterals during the study period. Third, the added procedure time and radiation exposure due to the additional collateral embolization were not assessed.

The present study is somewhat limited by the small sample size and the feasibility of selective catheterization of collaterals supplying the GS. However, to the best of our knowledge, this is the first study to report on technical aspects as well as clinical outcome of embolization of non-gonadal collateral supplying GS in addition to UAE, followed by hysteroscopic curettage for the management of CSP.

## Conclusions

During the management of UAE combined with hysteroscopic curettage for CSP, additional embolization of non-gonadal collateral supplying GS during UAE is feasible and safe in patients with non-gonadal collateral supplying GS, and the additional embolization of the collateral may reduce blood bloss related to hysteroscopic curettage.

## Data Availability

The datasets used and analysed during the current study are not publicly available, but are available from the corresponding author on reasonable request.

## Abbreviations

CSP: Cesarean Scar Pregnancy
FCE: Failed Collateral Embolization
GS: Gestational Sac
IIA: Internal Iliac Artery
SCE: Successful Collateral Embolization
UAE: Uterine Artery Embolization
β-hCG: β-human Chorionic Gonadotropin
